# Heart Team Intervention for Calcified Left Main Coronary Disease and Jeopardized Left Internal Mammary Artery Graft

**DOI:** 10.1155/2022/7712888

**Published:** 2022-06-22

**Authors:** Nobunari Tomura, Masashi Fujino, Yu Kataoka, Shuichi Yoneda, Hiroaki Sasaki, Teruo Noguchi

**Affiliations:** ^1^Department of Cardiovascular Medicine, National Cerebral and Cardiovascular Center, 6-1 Kishibe-Shimmachi, Suita, Osaka, Japan; ^2^Department of Cardiovascular Surgery, National Cerebral and Cardiovascular Center, 6-1 Kishibe-Shimmachi, Suita, Osaka, Japan

## Abstract

It is sometimes difficult to identify the culprit lesion and treatment strategy in patients with acute coronary syndrome who have complex coronary lesions and jeopardized left internal mammary artery graft. This report describes a heart team approach for a non-ST-segment elevation myocardial infarction case with complex coronary vasculature. A 73-year-old man presented to the emergency department with crescendo angina. He had a history of total aortic arch replacement with concomitant coronary artery bypass graft using left internal mammary artery. Emergent coronary angiography demonstrated severe stenosis at left main trunk bifurcation caused by calcified nodule. While the bypass graft to left anterior descending coronary artery was patent, the proximal segment of left subclavian artery was occluded. Following the prompt discussion with our heart team, we performed percutaneous coronary intervention in the first step for treating the left main stenosis using rotational atherectomy into the unprotected left circumflex artery. After clinical recovery, stress myocardial scintigraphy identified the presence of anteroseptal ischemia, which indicated coronary subclavian steal syndrome due to left subclavian artery occlusion. Contrast-enhanced CT visualized that the occlusion originated from the anastomosis, suggesting the potential procedural risk of endovascular treatment by dilatation. Our heart team discussed again and decided to undergo axillo-axillary artery bypass surgery. He was discharged 8 days after the surgery without any sequelae. This is the rare case report of non-ST-segment elevation myocardial infarction who had similar condition to coronary subclavian steal syndrome after total aortic arch replacement. This case highlights the importance of a collaborative approach of the heart team to identify the best therapeutic strategy in a patient with complex coronary vasculature.

## 1. Introduction

It is sometimes difficult to identify the culprit lesion and appropriate treatment strategy in patients with acute coronary syndrome who undergo coronary artery bypass grafting (CABG). A previous study of percutaneous coronary intervention (PCI) in patients with prior CABG demonstrated that the target vessel was a native coronary artery in 73.4% of cases and a bypass graft in the remaining 26.6% (saphenous vein graft, 25.0%; arterial graft, 1.5%) [1]. The coronary subclavian steal syndrome (CSSS) was first described in 1974 and is caused by retrograde or insufficient blood flow through the internal mammary artery graft, with subsequent myocardial ischemia. Proximal atherosclerotic stenosis of the ipsilateral subclavian artery is the most frequent cause of CSSS, and its consequences include angina or acute coronary syndrome [2, 3]. Although one case report discussed the endovascular treatment of CSSS complicated with coronary artery disease [4], the current case report describes the rare occurrence of non-ST-segment elevation myocardial infarction (NSTEMI) after total aortic arch replacement (TAR) in which a jeopardized left internal mammary artery (LIMA) graft resulted in a condition similar to CSSS.

## 2. Case Presentation

A 73-year-old man presented to the emergency department with crescendo angina. The patient had a past medical history of hypertension and diabetes mellitus. Four years earlier, he had undergone TAR with concomitant CABG using the LIMA to treat a thoracic aortic aneurysm and severe stenosis of the left anterior descending artery (LAD). The branch of the trifurcated graft for aortic arch replacement was sutured end-to-end to the left subclavian artery (LSA), and the LIMA was anastomosed to the LAD.

**Figure 1:**
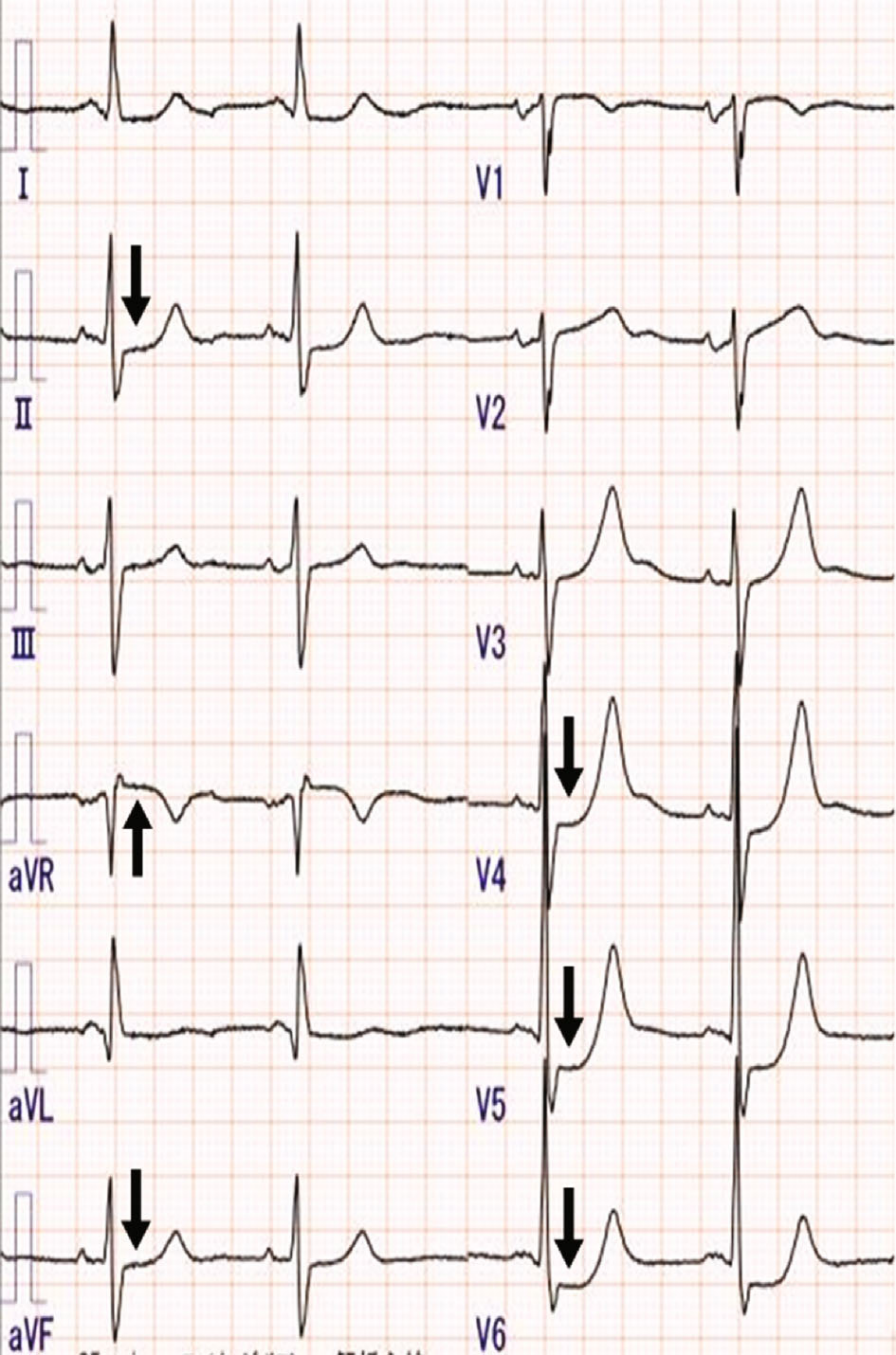
Electrocardiogram. Electrocardiogram showed ST-segment depression in leads II, aVF, and V4-V6 and ST-segment elevation in lead aVR.

On admission, he had a blood pressure of 117/75 mmHg, pulse rate of 66 beats/min, and arterial oxygen saturation of 97%. Physical examination was unremarkable, with no cardiac murmurs, respiratory sounds, or facial or leg edema. Chest X-ray revealed no pulmonary edema or cardiomegaly. Electrocardiography demonstrated ST-segment depression in leads II, aVF, and V4-V6 and ST-segment elevation in lead aVR (Figure 1). Echocardiography showed hypokinetic posterior wall motion (left ventricular ejection fraction 55%). The troponin T level was 2.45 ng/ml (reference < 0.014 ng/ml). We diagnosed the patient with NSTEMI. Despite the fact that he had a moderate GRACE risk score of 135 points, we performed emergent coronary angiography due to his medical history and electrocardiography results.

**Figure 2:**
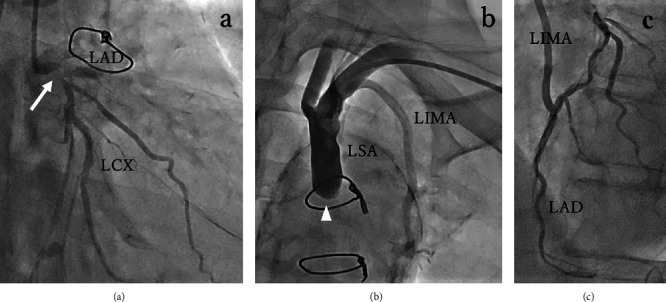
Angiography. Coronary angiography showed severe stenosis with a calcified nodule (arrow) at the left main coronary bifurcation (a). Subclavian angiography revealed occlusion of the proximal subclavian artery (arrowhead) (b). A left internal mammary artery bypass to the left anterior descending coronary artery was patent (c). LAD: left anterior descending artery; LCX: left circumflex artery; LIMA: left internal mammary artery; LSA: left subclavian artery.

Coronary angiography revealed severe stenosis with a calcified nodule at the left main bifurcation (Figure 2(a)). The proximal segment of the LSA was occluded (Figure 2(b)), although the LIMA bypass to the LAD was patent (Figure 2(c)). Angiography indicated myocardial ischemia in the region of the unprotected left circumflex artery (LCX), as well as suspected insufficient supply in the region of the LAD with concomitant LSA occlusion, a condition that resembled CSSS.

Our heart team of cardiovascular interventionists and surgeons promptly discussed potential treatments. PCI with a two-stent strategy for the calcified left main bifurcation lesion was thought to have potentially worse clinical outcomes than a one-stent strategy in terms of target lesion revascularization and stent thrombosis. The risk of re-CABG was estimated to be high, since the Society of Thoracic Surgeons risk score for mortality was 10.1%. Our heart team decided to perform PCI from the left main lesion to the LCX using a one-stent strategy and to then discuss elective revascularization of the occluded LSA.

**Figure 3:**
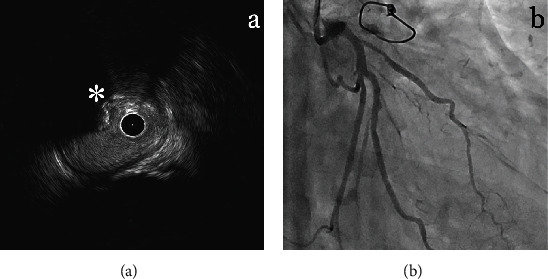
Intravascular imaging and final coronary angiography. Intravascular ultrasonography after rotational atherectomy revealed the presence of a calcified nodule (asterisk) (a). Final coronary angiography revealed TIMI3 flow to the left circumflex coronary artery after deployment of a drug-eluting stent.

We performed the ad hoc PCI for the lesions from the left main stenosis to the LCX. While the guidewire crossed successfully, an intravascular ultrasound (IVUS) catheter did not due to severe calcification. After balloon angioplasty with a 1.0 mm balloon, debulking was performed using a 1.5 mm rotational atherectomy burr, allowing subsequent IVUS catheter passage. IVUS images revealed a calcified nodule (Figure 3(a)). After additional balloon angioplasty with a 3.0 mm noncompliant balloon, a 3.5/28 mm sirolimus-eluting stent (Ultimaster, Terumo, Tokyo, Japan) was successfully implanted (Figure 3(b)).

**Figure 4:**
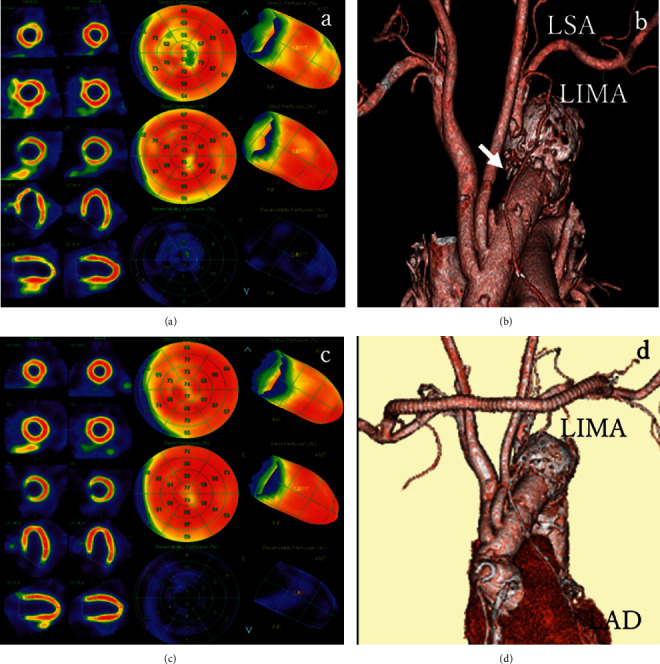
Myocardial scintigraphy and contrast-enhanced CT. Stress myocardial scintigraphy 7 days after the PCI procedure showed anteroseptal ischemia (a). Contrast-enhanced CT confirmed left subclavian artery occlusion at the anastomosed site (arrow) (b). Stress myocardial scintigraphy 3 days after axillo-axillary artery bypass surgery revealed improvement of myocardial ischemia (c). Contrast-enhanced CT 21 days postoperatively showed patency of the bypass graft (d). LAD: left anterior descending artery; LIMA: left internal mammary artery; LSA: left subclavian artery.

Stress myocardial scintigraphy performed 7 days after PCI identified anteroseptal ischemia (Figure 4(a)), which indicated CSSS due to LSA occlusion. Contrast-enhanced CT visualized LSA occlusion at the anastomosed site (Figure 4(b)), suggesting procedural risk associated with endovascular treatment. Our heart team decided to perform axillo-axillary artery bypass surgery.

Stress myocardial scintigraphy performed 3 days postoperatively showed an improvement in anteroseptal ischemia (Figure 4(c)). The patient was discharged without chest pain 8 days postoperatively. Contrast-enhanced CT performed 21 days postoperatively showed patency of the bypass graft (Figure 4(d)).

## 3. Discussion

We experienced a case of NSTEMI with complex left main coronary lesions and a jeopardized LIMA graft after TAR. There were three main challenges: identifying the culprit lesion; determining the optimal PCI treatment of the left main coronary bifurcation lesion with the calcified nodule; and deciding how to treat the jeopardized LIMA graft after TAR, which resulted in a condition that resembled CSSS. Our heart team performed emergent PCI for the lesions from the left main stenosis to the LCX and conducted elective surgery for the LSA occlusion to treat the jeopardized LIMA graft.

Although it was difficult to identify the culprit lesion, we chose the optimal treatment from among three options: re-CABG, PCI for the left main bifurcation lesion with a two-stent strategy, or hybrid PCI therapy for the lesions from the left main stenosis to the LCX and revascularization for the occluded LSA. Re-CABG was estimated to carry high surgical risk. PCI for a calcified nodule is challenging despite advances in coronary intervention procedures. Previously, we reported that acute coronary syndrome attributable to a calcified nodule was associated with an increased risk of target lesion revascularization [5]. Furthermore, the bifurcation two-stent strategy was associated with higher rates of target lesion revascularization and stent thrombosis than the one-stent strategy [6]. Since angiography suggested that the LSA was chronically occluded, a single-stent strategy for the left main bifurcation lesion with the calcified nodule was deemed preferable.

The American College of Cardiology (ACC) guidelines state that revascularization using the ipsilateral internal mammary artery is reasonable in asymptomatic patients with subclavian artery stenosis (Class IIa) when performed either by extra-anatomic bypass surgery or subclavian angioplasty and stenting [7]. The technical success rate of the percutaneous approach can be >90%, with 5-year patency rates of 85% [8]. Surgical revascularization has a patency rate in excess of 70% at 5 years [9]. Since there were concerns about procedural risks of endovascular treatment for LCA occlusion at the anastomosed site, our heart team prioritized surgical treatment.

It is important that complex coronary artery disease be treated following meticulous evaluation of target lesions by a heart team. In this case, our heart team successfully performed emergent PCI for left main stenosis with a one-stent strategy, as well as selective surgery for the jeopardized LIMA graft. The ACC expert consensus document on PCI without on-site surgery also states that patients with high-risk lesions should not undergo nonemergent PCI at facilities without the capacity for on-site surgical procedures [10]. We should immediately collaborate with surgeons even when we treat such patients at these facilities.

## Data Availability

All relevant data supporting the conclusions of this article are included within the article.

## Abbreviations

ACC: American College of Cardiology
CABG: Coronary artery bypass grafting
CSSS: Coronary subclavian steal syndrome
IVUS: Intravascular ultrasound
LAD: Left anterior descending artery
LCX: Left circumflex artery
LIMA: Left internal mammary artery
LSA: Left subclavian artery
NSTEMI: Non-ST-segment elevation myocardial infarction
PCI: Percutaneous coronary intervention
TAR: Total aortic arch replacement.

## Supplementary Materials

Supplementary Materials Care checklist of information to include when writing a case report.
