# Supplementary material for: Heart Team Intervention for Calcified Left Main Coronary Disease and Jeopardized Left Internal Mammary Artery Graft
Source: Case Rep Cardiol. 2022 Jun 22;2022:7712888. doi: 10.1155/2022/7712888 (PMC9242785; doi:10.1155/2022/7712888)
Supplement: Supplementary Materials — Care checklist of information to include when writing a case report. [file 7712888.f1.pdf]

CARE case report guidelines

CARE Checklist of information to include when writing a case report

(cc) BY-NC-ND

✓

Topic	Item	Checklist item description	Reported on Line
**Title**	1	The diagnosis or intervention of primary focus followed by the words "case report"	Paragraph 2
**Key Words**	2	2 to 5 key words that identify diagnoses or interventions in this case report, including "case report"	Paragraph 39-40
**Abstract (no references)**	3a	Introduction: What is unique about this case and what does it add to the scientific literature?	Paragraph 58-60
	3b	Main symptoms and/or important clinical findings	Paragraph 45-50
	3c	The main diagnoses, therapeutic interventions, and outcomes	Paragraph 51-58
	3d	Conclusion—What is the main "take-away" lesson(s) from this case?	Paragraph 60-62
**Introduction**	4	One or two paragraphs summarizing why this case is unique (**may include references**)	Paragraph 74-75
**Patient Information**	5a	De-identified patient specific information.	Paragraph 79
	5b	Primary concerns and symptoms of the patient.	Paragraph 79
	5c	Medical, family, and psycho-social history including relevant genetic information	Paragraph 80
	5d	Relevant past interventions with outcomes	Paragraph 80-84
**Clinical Findings**	6	Describe significant physical examination (PE) and important clinical findings.	Paragraph 85-87
**Timeline**	7	Historical and current information from this episode of care organized as a timeline	N/A
**Diagnostic Assessment**	8a	Diagnostic testing (such as PE, laboratory testing, imaging, surveys).	Paragraph 85-91
	8b	Diagnostic challenges (such as access to testing, financial, or cultural)	N/A
	8c	Diagnosis (including other diagnoses considered)	Paragraph 91
	8d	Prognosis (such as staging in oncology) where applicable	N/A
**Therapeutic Intervention**	9a	Types of therapeutic intervention (such as pharmacologic, surgical, preventive, self-care)	Paragraph 118
	9b	Administration of therapeutic intervention (such as dosage, strength, duration)	Paragraph 131
	9c	Changes in therapeutic intervention (with rationale)	Paragraph 134-135
**Follow-up and Outcomes**	10a	Clinician and patient-assessed outcomes (if available)	N/A
	10b	Important follow-up diagnostic and other test results	Paragraph 136-137
	10c	Intervention adherence and tolerability (How was this assessed?)	Paragraph 137-139
	10d	Adverse and unanticipated events	N/A
**Discussion**	11a	A scientific discussion of the strengths AND limitations associated with this case report	Paragraph 173-179
	11b	Discussion of the relevant medical literature **with references**.	Paragraph 155-172
	11c	The scientific rationale for any conclusions (including assessment of possible causes)	Paragraph 155-172
	11d	The primary "take-away" lessons of this case report (without references) in a one paragraph conclusion	Paragraph 173
**Patient Perspective**	12	The patient should share their perspective in one to two paragraphs on the treatment(s) they received	Paragraph 137
**Informed Consent**	13	Did the patient give informed consent? Please provide if requested	Yes ☑ No ☐
